# Sirtuins and Insulin Resistance

**DOI:** 10.3389/fendo.2018.00748

**Published:** 2018-12-06

**Authors:** Shuang Zhou, Xiaoqiang Tang, Hou-Zao Chen

**Affiliations:** ^1^Internal Medicine, Peking Union Medical College Hospital, Beijing, China; ^2^Key Laboratory of Birth Defects and Related Diseases of Women and Children of MOE, State Key Laboratory of Biotherapy, West China Second University Hospital, Sichuan University, Chengdu, China; ^3^State Key Laboratory of Medical Molecular Biology, Department of Biochemistry and Molecular Biology, Institute of Basic Medical Sciences, Chinese Academy of Medical Sciences & Peking Union Medical College, Beijing, China

**Keywords:** sirtuins, insulin resistance, senescaging, inflammation, mitochondrial dysfunction

## Abstract

The mammalian Sirtuins (SIRT1-7) are an evolutionarily conserved family of NAD^+^-dependent deacylase and mono-ADP-ribosyltransferase. Sirtuins display distinct subcellular localizations and functions and are involved in cell survival, senescence, metabolism and genome stability. Among the mammalian Sirtuins, SIRT1 and SIRT6 have been thoroughly investigated and have prominent metabolic regulatory roles. Moreover, SIRT1 and SIRT6 have been implicated in obesity, insulin resistance, type 2 diabetes mellitus (T2DM), fatty liver disease and cardiovascular diseases. However, the roles of other Sirtuins are not fully understood. Recent studies have shown that these Sirtuins also play important roles in inflammation, mitochondrial dysfunction, and energy metabolism. Insulin resistance is the critical pathological trait of obesity and metabolic syndrome as well as the core defect in T2DM. Accumulating clinical and experimental animal evidence suggests the potential roles of the remaining Sirtuins in the regulation of insulin resistance through diverse biological mechanisms. In this review, we summarize recent advances in the understanding of the functions of Sirtuins in various insulin resistance-associated physiological processes, including inflammation, mitochondrial dysfunction, the insulin signaling pathway, glucose, and lipid metabolism. In addition, we highlight the important gaps that must be addressed in this field.

## Introduction

The increasing prevalence of obesity and associated metabolic syndrome (including type 2 diabetes mellitus [T2DM], nonalcoholic fatty liver disease [NAFLD], atherosclerosis and atherosclerotic heart disease) is an increasingly severe challenge in public health (1). Insulin resistance is the critical, universal pathological feature of these diseases, especially T2DM. Insulin is a major hormone secreted by pancreatic β cells after nutrient stimulation and plays a critical role in reducing blood glucose concentration by facilitating glucose uptake by skeletal muscle and adipose tissue and inhibiting endogenous glucose production in the liver (2–4). Insulin resistance occurs when cells are incapable of efficiently responding to a normal dose of insulin (2–4). The development of insulin resistance is a multistep, complex process influenced by genetics and environments (3). Although the precise pathogenesis of insulin resistance remains incompletely understood, several mechanisms are proposed to be involved, including defects in the insulin signaling pathway, ectopic lipid accumulation, systemic inflammation, mitochondrial dysfunction, oxidative stress, and endoplasmic reticulum (ER) stress, as reviewed elsewhere (1, 5, 6).

The mammalian Sirtuins are a family of NAD^+^-dependent deacetylases (7). This family consists of seven members (SIRT1-SIRT7), which share the conserved Sirtuin domain conferring NAD^+^-dependent deacetylase activity but have variable amino- and carboxy-terminal extensions and display distinct subcellular localization and biological functions (8, 9). SIRT1 is mainly localized to the nucleus (10), but it shuttles between the nucleus and cytoplasm during development and in response to physiological and pathological stress (11). In contrast to SIRT1, mammalian SIRT2 is mainly localized to the cytoplasm (10, 12) but is also found in the nucleus (13). SIRT3, SIRT4, and SIRT5 are localized to mitochondria (10, 14), whereas SIRT6 and SIRT7 are found in the nucleus (10). SIRT6 is a chromatin-associated protein, and SIRT7 resides in the nucleolus (15, 16). Deacetylase activity was initially reported as conserved in mammalian Sirtuins, but different Sirtuins exhibit different acyl group preferences (17). Among the seven Sirtuins, SIRT4-7 exhibit weak or undetectable deacetylation activity *in vitro* (10, 18). SIRT2 reportedly possesses efficient demyristoylase activity (18). SIRT5 is reportedly an efficient NAD^+^-dependent protein, lysine desuccinylase and demalonylase (19), while SIRT4 and SIRT6 reportedly possess ADP-ribosyltransferase activity (20, 21).

Mammalian Sirtuins regulate a wide variety of cellular processes, including metabolism, mitochondrial homeostasis, oxidative stress, inflammation, autophagy and apoptosis (22). Sirtuins also play important roles in aging and aging-related diseases, such as obesity, T2DM, cardiovascular disease, cancer, neurodegenerative diseases (23). SIRT1 and SIRT6 are the most extensively characterized Sirtuins. A large body of literature indicates that these two Sirtuins play an important role in metabolism, and they have also recently attracted increased attention with regard to their protective roles in maintaining insulin sensitivity, as reviewed elsewhere (24–26). Compared to the metabolic roles of SIRT1 and SIRT6, the metabolic roles of other Sirtuins remain poorly understood. Here, we review the recent advances in the understanding of the roles of Sirtuins in inflammation, mitochondrial dysfunction, and oxidative stress and discuss their possible roles in insulin resistance.

## Sirtuins in Inflammation

Obesity-induced chronic, low-grade inflammation is one of the most important contributors to the pathogenesis of insulin resistance (27–29). Adipose tissue is not only an insulin-targeting organ for lipid metabolism but also an endocrine organ that secretes hormones, cytokines, and chemokines to influence insulin sensitivity. For instance, adipocytes secrete adipokines, such as leptin and adiponectin, to promote insulin sensitivity (30, 31), and resistin and retinol-binding protein 4 (RBP4) to impair insulin sensitivity (32, 33). Importantly, adipose tissue is a critical initiator of the inflammatory response to obesity (28). In obesity, metabolism and gene expression of adipocytes change, resulting in increased lipolysis of adipocytes, the release of free fatty acids and proinflammatory cytokines and activation of M1 macrophages (27–29). M1 macrophages produce a large number of proinflammatory mediators, such as TNF-α, IL-1β, and resistin, that act on adipocytes to induce an insulin-resistant state and activate inflammatory pathways in insulin-targeting cells. Ultimately, ectopic lipid deposition and increased expression of inflammatory mediators in the liver and skeletal muscle lead to impaired insulin signaling and exacerbate systemic insulin resistance (29). Signals from all the proinflammatory mediators converge on inflammatory signaling pathways, including jun-n-terminal kinase (JNK) and inhibitor of nuclear factor κB (NF-κB) kinase (IKK) (27, 34, 35). Inhibition of insulin receptor downstream signaling is the primary mechanism for inflammation-induced insulin resistance (27). Activated JNK or IKK can phosphorylate the insulin receptor (IR) and insulin receptor substrate (IRS) proteins and decrease their tyrosine phosphorylation, thus leading to decreased activation of PI3-kinase and Akt and resistance to the metabolic actions of insulin (34, 36). In addition, activation of the JNK and IKK pathways can induce the production of inflammatory mediators, while the Sirtuin family plays essential roles in inflammation, which comprehensively contributes to insulin resistance.

### Inflammatory Transcriptional Factor

NF-κB is a key transcriptional factor that mediates the expression of multiple inflammatory factors, including TNF-α, IL-1β, and IL-6. The acetylation of NF-κB promotes its nuclear translocation and activation. SIRT1 has been demonstrated to repress inflammation in multiple tissues and cells (37–40). In particular, SIRT1 suppresses inflammation in both adipocytes (39, 41) and macrophages (42), which leads to a reduction of adipose tissue inflammation. SIRT1 deacetylates p65 subunit of NF-κB at lysine 310 (K310) and inhibits the transcriptional activity of NF-κB (43). Moreover, SIRT1 interacts with transducing-like enhancer of split 1 (TLE1), a co-repressor of NF-κB, to inhibit NF-κB-mediated transcription (44). In addition, SIRT1 deacetylates activator protein-1 (AP-1) to reduce the expression of COX-2 in macrophages and deacetylates p53 to repress macrophage activation (45, 46). Similar to SIRT1, SIRT2 also binds to NF-κB and mediates the deacetylation of NF-κB subunit p65 at K310, which leads to the inhibition of the expression of NF-κB target inflammatory genes in fibroblasts, macrophages and microglial cells (47–49). SIRT2-mediated inhibition of NF-κB and inflammation contributes to its anti-inflammatory function in an experimental colitis mouse model (49), neuroinflammation (48, 50, 51), collagen-induced arthritis (52), and microvascular inflammation in *ob/ob* septic mice (53). In addition to SIRT2, SIRT4 can regulate the activation of NF-κB. SIRT4 has been shown to negatively regulate cigarette smoke extract (CSE)-induced NF-κB activation by inhibiting the degradation of IκBα and inhibiting NF-κB target gene expression, including the proinflammatory cytokines IL-1β, TNF-α, and IL-6, resulting in inhibition of CSE-induced mononuclear cell adhesion to human pulmonary microvascular endothelial cells (54). SIRT4 can prevent NF-κB nuclear translocation as well as the transcriptional activity of NF-κB, thereby suppressing inflammation in human umbilical vein endothelial cells (55). Interestingly, the role of SIRT5 in inflammation is controversial. Recently, Qin and colleagues showed that SIRT5 deficiency decreased toll-like receptor (TLR)-triggered inflammation in both acute and immunosuppressive phases of sepsis (56). Mechanistically, SIRT5 competes with SIRT2 to interact with NF-κB p65 in a deacetylase activity-independent manner and thus blocks the deacetylation of p65 by SIRT2, which consequently leads to the activation of the NF-κB pathway and induction of its downstream cytokines in macrophages (56). However, Wang and colleagues found that SIRT5 desuccinylates and actives pyruvate kinase isoform M2 (PKM2) by promoting its dimerization and nuclear accumulation, thereby decreasing proinflammatory cytokine IL-1β production in LPS-activated macrophages (57). As hyperproduction of IL-1β contributes to increased susceptibility to inflammatory bowel disease, *Sirt5-*deficient mice are more susceptible to dextran sulfate sodium (DSS)-induced colitis (57). Interestingly, *Sirt6* deficient mice display increased expression of NF-κB-dependent genes in multiple tissues (58). *Sirt6* deletion increases inflammation in the mice adipose tissue and promotes HFD-induced insulin resistance (59, 60). Mechanistically, SIRT6 binds to the NF-κB subunit RelA and deacetylates histone H3 lysine 9 (H3K9) at NF-κB target gene promoters, which leads to a reduction of NF-κB target gene expression (58). These findings suggest that the Sirtuins target inflammatory transcriptional factors (e.g., NF-κB and AP1) directly or indirectly to contribute to insulin resistance comprehensively.

### Inflammasome

The anti-inflammatory role of Sirtuins involves other mechanisms. The Nod-like receptor family, pyrin domain-containing 3 (NLRP3) inflammasome is a multiprotein complex that orchestrates the innate immune responses of macrophages by controlling the activation of caspase-1 and the release of the proinflammatory cytokines IL-1β and IL-18 (61–63). Obesity-related inflammation is partly mediated by the NLRP3 inflammasome, and NLRP3 activation exacerbates obesity-linked diseases (64, 65). Resveratrol, a SIRT1 activator, inhibits ionizing irradiation-induced inflammation in mesenchymal stem cells *via* suppressing NLRP3 inflammasome activation (66). In a murine model of sepsis, *Sirt1* deletion results in increasing lung inflammasome activation and inflammatory lung injury (67). A recent study demonstrated that silybin prevents NLRP3 inflammasome activation during NAFLD through SIRT2 (68). However, further studies are needed to clarify the mechanism underlying SIRT2-mediated regulation of NLRP3 inflammasome activity. In a human fasting/refeeding study, Traba and colleagues observed that fasting leads to a reduction in NLRP3 inflammasome activation (69). SIRT3 deletion in a human macrophage line increases NLRP3 inflammasome activation, accompanied by excessive mitochondrial ROS production (69). Pharmacologic and genetic SIRT3 activation enhances mitochondrial function and suppresses NLRP3 activity in THP-1 monocyte cells and in leukocytes extracted from healthy volunteers and from refeeding individuals (69). The authors concluded that nutrient levels regulate the NLRP3 inflammasome partly through SIRT3-mediated mitochondrial homeostatic control. Similarly, Chen *et al*. reported that trimethylamine-N-oxide (TMAO) increases ROS production by inhibiting the SIRT3-SOD2-mitochondrial signaling pathway, which leads to NLRP3 inflammasome activation and consequently promotes vascular inflammation (70). Defective autophagy in monocytes or macrophages might result in NLRP3 inflammasome activation and cause vascular metabolic inflammation (71–73). Acetylation of ATG5, an autophagy-related protein, inhibits autophagosome maturation and induces NLRP3 inflammasome activation (74). Recently, Liu and colleagues demonstrated that SIRT3 binds with ATG5 and deacetylates it, while SIRT3-deficient macrophages display impaired autophagy, leading to accelerated NLRP3 inflammasome activation and endothelial dysfunction (73). These studies suggest that SIRT3 may inhibit NLRP3 inflammasome activation by regulating mitochondrial function, ROS production, and autophagy. As SIRT2 has also been shown to regulate NLRP3 inflammasome activation (68), the potential synergistic effect on regulation of NLRP3 inflammasome activation between SIRT3 and SIRT2 needs further study. Previous studies have highlighted the anti-inflammatory role of SIRT3 in obesity-related diseases, including insulin resistance. The function of SIRT3 in inflammasome regulation largely depends on SIRT3-mediated activation of MnSOD and suppression of oxidative stress. These findings implicate that the SIRT1-SIRT3 indirectly regulate the activation of the NLRP3 inflammasome, which may be involved in the modulation of insulin resistance. However, whether Sirtuins directly regulate inflammasome remains unknown.

### Sirtuins, Inflammation and Insulin Resistance

The roles of Sirtuins in inflammation significantly contribute to their functions during insulin resistance. For instance, activation of SIRT1 leads to the repression of JNK and IKK inflammatory pathways greatly and subsequently improves glucose tolerance, reduced hyperinsulinemia, and enhanced systemic insulin sensitivity (40). SIRT1 also controls the inflammatory status of macrophages and T lymphocytes to regulate the metabolism (insulin sensitivity) and inflammation of adipose tissues in obese mice (41, 75–77). In addition, SIRT6 is important for macrophage activation and TNFα production (78). Myeloid *Sirt6* deficiency causes insulin resistance in HFD–fed mice by eliciting macrophage polarization toward an M1 phenotype (79), and facilitates the development of HFD-induced atherosclerosis (80). Deletion of *Sirt6* in T cells or myeloid-derived cells is sufficient to induce liver inflammation and fibrosis (81). Interestingly, SIRT1 and SIRT6 can coordinate a switch from glucose to fatty acid oxidation during the acute inflammatory response (82). Therefore, Sirtuins not only regulate the inflammatory pathways within the target cells (e.g., hepatocytes, skeletal muscle cells, adipocytes) to affect their insulin sensitivity but also regulate inflammatory cells infiltrated in the organs, where the Sirtuins respond to inflammatory and metabolic insults and subsequently regulate insulin sensitivity and disease by targeting inflammatory cell activation and differentiation. Notably, the Sirtuins may also cooperate in diverse types of cells or within the same type of cell during inflammation-associated insulin resistance.

## Sirtuins in Mitochondrial Dysfunction

Mitochondria are the primary site for ATP generation and ROS production. Mitochondrial dysfunction results in decreased ATP production, increased ROS production and accumulated mitochondrial DNA damage, which contribute to insulin resistance (5). Cells eliminate ROS by expressing endogenous antioxidant enzymes, including manganese superoxide dismutase (MnSOD), catalase, glutathione peroxidase (GPX) and glutathione reductase (GRx) (83, 84). An imbalance between the production of ROS and antioxidant enzymes leads to oxidative stress, which has been implicated in the pathogenesis of insulin resistance, obesity, and diabetes (1, 85).

### SIRT1

There are fewer mitochondria in muscles of T2DM patients than those of insulin-sensitive individuals (86). Marked reduction of oxidative phosphorylation in the mitochondria can be detected in the liver and skeletal muscle of T2DM patients and insulin-resistant individuals (87, 88). Peroxisome proliferator-activated receptor gamma coactivator 1-alpha (PGC-1α) is a transcriptional coactivator that regulates mitochondrial biogenesis and respiration (89, 90). SIRT1 has been shown to deacetylate PGC-1α at several lysine residues and regulates PGC-1α activity, which activates the transcription of genes involved in mitochondrial biogenesis (91). SIRT1 activator resveratrol promotes PGC-1α activity and increases the number of mitochondria in muscle cells, which improves mitochondrial function and protects mice against diet-induced obesity and insulin resistance (92). In addition to mitochondria biogenesis, SIRT1 regulates mitochondrial function through clearance of damaged mitochondria (93). Mechanistically, SIRT1 binds to and deacetylates autophagy regulators (including ATG5, ATG7, and ATG8) to induce mitochondria autophagy or mitophagy (93). SIRT1-mediated deacetylation of FoxO1 and FoxO3a is also known to induce the expression of autophagy pathway components (94, 95). Accumulating evidence has shown that SIRT1 safeguards cells from oxidative stress. SIRT1 reduces ros production by deacetylating and activating FoxO3a to upregulate expression of MnSOD and catalase (95, 96). SIRT1 promotes the transcriptional activity of Nuclear factor (erythroid-derived 2)-like 2 (NRF2) by deacetylating it and upregulates the expression of NRF2 target antioxidant genes, including *MnSOD, catalase, glutathione*, and *heme oxygenase-1* (*HO-1*) (97).

### SIRT2

Oxidative stress increases SIRT2 expression *in vivo* and *in vitro* (98, 99). SIRT2 can bind to FoxO3a and deacetylate it, leading to an increase in FoxO3a transcriptional activity, upregulation of the expression of FoxO target genes such as *MnSOD, Bim*, and *p27*^*Kip*1^, and a consequent decrease in ROS generation (98). Nicotinamide adenine dinucleotide phosphate (NADPH) is a functionally important metabolite that is required to generate the reduced form of glutathione (GSH) to maintain cellular redox potential. In response to oxidative stimuli, SIRT2 deacetylates and activates glucose 6-phosphate dehydrogenase (G6PD), a key enzyme in the pentose phosphate pathway (PPP), resulting in increased cytosolic NADPH to attenuate oxidative damage (100). Similarly, oxidative stress increases the glycolytic enzyme phosphoglycerate mutase (PGAM)-SIRT2 interaction, leading to deacetylation and activation of PGAM, which increases the cellular NADPH level to counteract oxidative damage (101). Lysine 4-oxononanoylation (4-ONylation) is a newly discovered histone posttranslational modification that disrupts the interaction between histone H3 and DNA, thereby preventing nucleosome assembly under oxidative stress (102). SIRT2 was reported to remove the 4-oxononanoyl (4-ONyl) lysine groups on histones and attenuate the negative impact of protein 4-ONylation caused by oxidative stress (103). This study provides novel evidence that SIRT2 may exert antioxidation effects through epigenetic modification. Oxidative stress is not completely caused by mitochondria, and whether SIRT2 can influence oxidative stress by regulating mitochondrial function is unclear.

Interestingly, a recent work by Liu and colleagues observed that SIRT2 can localize to the inner mitochondrial membrane of mouse central nervous system cells and that the acetylation of several metabolic mitochondrial proteins is altered in *Sirt2*-deficient mice. In mice, deletion of *Sirt2* causes mitochondrial morphological changes, increases oxidative stress and decreases ATP production in MEFs and brain tissues (104), indicating that SIRT2 may deacetylate antioxidant enzymes in the mitochondria directly.

### SIRT3

SIRT3, a central mitochondrial deacetylase, deacetylates, and activates mitochondrial enzymes to regulate mitochondrial metabolism, oxidative stress, cell survival, and longevity (105). SIRT3 has been shown to play a pivotal role in maintaining mitochondrial function and ROS homeostasis. For example, SIRT3 deacetylates complex I and complex II of the electron transport chain to promote electron transport and regulate energy homeostasis (106, 107). SIRT3 deacetylates cyclophilin D (CypD), the regulatory component of the mitochondrial permeability transition pore (mPTP), preventing opening of the mPTP and mitochondrial dysfunction (108). SIRT3 binds to and deacetylates 8-oxoguanine-DNA glycosylase 1 (OGG1), a DNA repair enzyme that excises 7,8-dihydro-8-oxoguanine from the damaged genome, resulting in the repair of mitochondrial DNA (mtDNA) damage, protection of mitochondrial integrity, defense against mitochondrial dysfunction and prevention of stress-induced cellular apoptosis (109). In contrast, *Sirt3* deficiency has been linked to increased ROS production (110, 111). The antioxidant action of SIRT3 involves MnSOD and mitochondrial isocitrate dehydrogenase 2 (IDH2). SIRT3 has been shown to block cardiac hypertrophy by deacetylating FOXO3a and upregulating the expression of FOXO3a target genes such as *MnSOD* and *catalase*, decreasing ROS generation (112). Notably, the antioxidative action of SIRT3 may also be attributed to its direct deacetylation and promotion of the enzyme activity of MnSOD. SIRT3 deacetylates two critical lysine residues (K53 and K89) of MnSOD that promote MnSOD antioxidation activity, thereby reducing cellular ROS (113). Direct deacetylation of lysine residues, including K68 and K122 by SIRT3, also increases the enzyme activity of MnSOD and decreases ROS production (114, 115). In addition to MnSOD, SIRT3 directly deacetylates and activates mitochondrial IDH2, which results in increased NADPH levels and an increased ratio of reduced to oxidized GSH in mitochondria, thus protecting cells from oxidative stress-induced damage (110, 116). These studies suggest that SIRT3 activity is necessary to prevent mitochondrial dysfunction and reduce oxidative stress.

### SIRT4

SIRT4 functions as an efficient mitochondrial ADP-ribosyl transferase that negatively impacts gene expression and various metabolic processes in mitochondria. Our previous work demonstrated that SIRT4 promotes angiotensin II-induced development of cardiac hypertrophy by inhibiting the interaction of SIRT3 and MnSOD, which increases MnSOD acetylation levels, decreases its activity and leads to elevated ROS accumulation (117). SIRT4 may play a role different from that of SIRT3 in regulating mitochondrial function and oxidative stress. *Sirt4* deficiency *in vivo* and *in vitro* increases the expression of genes involved in fatty acid β-oxidation and oxidative phosphorylation, thus enhancing fatty acid oxidation and mitochondrial respiration in liver and muscle (118, 119). SIRT4 increases stress-induced mitochondrial ROS production and interacts with the long form of GTPase optic atrophy 1 (L-OPA1) to promote mitochondrial fusion, thereby inhibiting mitophagy and decreasing the removal of dysfunctional mitochondria (120).

### SIRT5

SIRT5 functions to deacetylate, demalonylate, and desuccinylate multiple proteins in mitochondria (19). SIRT5 is involved in the regulation of mitochondrial fatty acid β-oxidation, the urea cycle, and cellular respiration (84, 121). SIRT5 binds to, desuccinylates and activates copper-zinc superoxide dismutase 1 (SOD1) to eliminate ROS (122). Moreover, SIRT5 desuccinylates IDH2 and deglutarylates G6PD, thus activating both NADPH-producing enzymes to scavenge ROS (123). SIRT5 also binds, desuccinylates and inhibits the activity of the glycolysis enzyme PKM2, which facilitates the diversion of glucose metabolites into the pentose phosphate shunt and then produces sufficient NADPH to eliminate ROS (123). Interestingly, a very recent study showed that SIRT5 is present in peroxisomes and can bind, desuccinylate and inhibit ACOX1, the first and rate-limiting enzyme in fatty acid β-oxidation and a major producer of H_2_O_2_, attenuating peroxisome-induced oxidative stress (124).

### SIRT6

SIRT6 plays an important role in DNA repair, genomic stability, and cellular senescence, however, the role of SIRT6 in oxidative stress has not been well clarified. According to recent studies, SIRT6 is believed to protect cells against oxidative stress. Pan et al. (125) found that SIRT6 serves as an NRF2 coactivator by interacting with NRF2 and RNA polymerase II to transactivate NRF2-regulated antioxidant genes, including HO-1. SIRT6 activates AMPK-FoxO3a axis to initiate expression of *MnSOD* and *catalase* and protects cardiomyocytes against ischemia/reperfusion-induced injury (126).

### SIRT7

*Sirt7* deficiency in mice induces multisystemic mitochondrial dysfunction (127). SIRT7 deacetylates GABPβ1, a master regulator of nuclear-encoded mitochondrial genes, enables it to form the transcriptionally active GABPa/GABPβ heterotetramer, and promotes mitochondria function (127). Additionally, the mitochondrial unfolded protein response [UPR(mt)] is mediated by the interplay of SIRT7 and NRF1 and is coupled to cellular energy metabolism and proliferation (128).

## Sirtuins in Insulin Signaling Pathways

### Insulin Secretion

**Figure 1:**
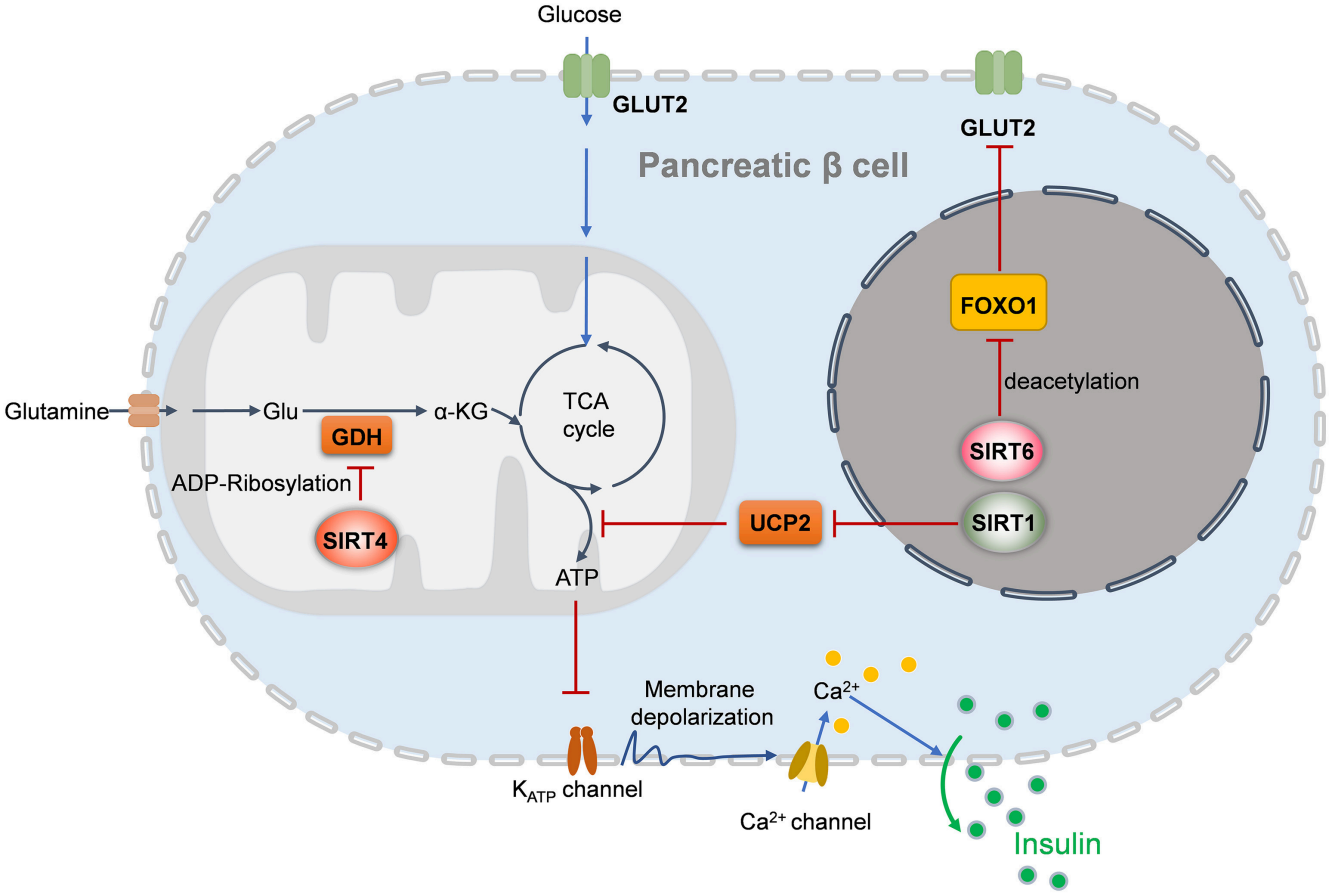
Sirtuins regulates insulin secretion of pancreatic beta cells. In the nucleus, SIRT1 induces insulin secretion through the reduction of UCP2 expression and the enhancement of depolarization in pancreatic β cells, while SIRT6 deacetylates FOXO1 and promotes the expression of GLUT2, which facilitates glucose uptake and insulin secretion. In the mitochondria, SIRT4 promotes the ADP-ribosylation and inactivation of GDH, leading the repression of ATP generation and inhibition of insulin secretion. UCP2, uncoupling protein 2; Glu, glutamate; GDH, glutamate dehydrogenase; GLUT2, glucose transporter 2; α-KG, alpha-ketoglutarate.

Under the condition of insulin resistance, normal pancreatic β cells increase the production of insulin to maintain blood glucose levels. However, this compensatory response fails, and relative insulin insufficiency develops. Then, glucose tolerance is impaired, and T2DM eventually occurs. SIRT1, SIRT4, and SIRT6 reportedly regulate pancreatic β cell function (Figure 1) (24, 129, 130). According to accumulating evidence, SIRT1 and SIRT6 repress pancreatic β cell dysfunction, attenuating the development of T2DM. β cell-specific SIRT1 transgenic mice exhibit enhanced insulin secretion and improved glucose tolerance to high glucose stimulation (131). Mechanistically, through repressing UCP2 expression, SIRT1 enhances ATP production in pancreatic β cells, to shut down the potassium channel, resulting in the influx of calcium and finally the secretion of insulin (131, 132). SIRT1 can induce *NeuroD* and *MafA* expression *via* deacetylating and activating FoxO1, which protects pancreatic β cells against oxidative damage and preserves pancreatic β cells function (133). In contrast to SIRT1 and SIRT6, SIRT4 functions as a negative regulator of insulin secretion in β cells. Glutamate dehydrogenase (GDH) promotes the metabolism of glutamate and glutamine, generating ATP to further promote insulin secretion. In pancreatic β cells, SIRT4 represses the activity of GDH by ADP-ribosylation, thereby downregulating insulin secretion in response to amino acids under calorie-sufficient conditions (20). SIRT4 also controls leucine oxidation to regulate insulin secretion (134). Given that SIRT3 deacetylases GDH and increases its activity in hepatocytes (135), SIRT3 may function in β cell mitochondria to promote insulin secretion. Recent studies have provided evidence to support this notion. Caton *et al*. observed that SIRT3 expression markedly decreases in islets isolated from T2DM patients, as well as in mouse islets or INS1 cells (136). *Sirt3* knockdown in INS1 cells results in increased production of cellular ROS and IL-1β, increased β cell apoptosis and reduced insulin secretion (136). SIRT3 deficiency predisposes pancreatic β cells to oxidative stress-induced dysfunction and reduces glucose-induced insulin secretion (137). By contrast, SIRT3 overexpression inhibits ER stress and attenuates palmitate-induced pancreatic β cell dysfunction (138, 139). Therefore, SIRT3 and SIRT4 play opposing roles in regulating insulin secretion in pancreatic β cells. In addition, insulin secretion impairment is observed in *Sirt6* knockout pancreatic β cells, which is mediated by suppression of the FoxO1-Pdx1-Glut2 pathway (140). *Sirt6* deletion in pancreatic β cells also reduces ATP production and increases mitochondrial damage which induces cell apoptosis and impairs glucose-stimulated insulin secretion (129, 141). β cell-specific *Sirt6-*ko mice are glucose intolerance and are defective in glucose-stimulated insulin secretion, in spite do not show abnormality in endocrine morphology, pancreatic β cell mass or insulin production (130). *Sirt6* deficiency also results in aberrant upregulation of thioredoxin-interacting protein (TXNIP) in pancreatic β cells, which inhibits insulin secretion (130).

### Insulin Signaling Pathway

**Figure 2:**
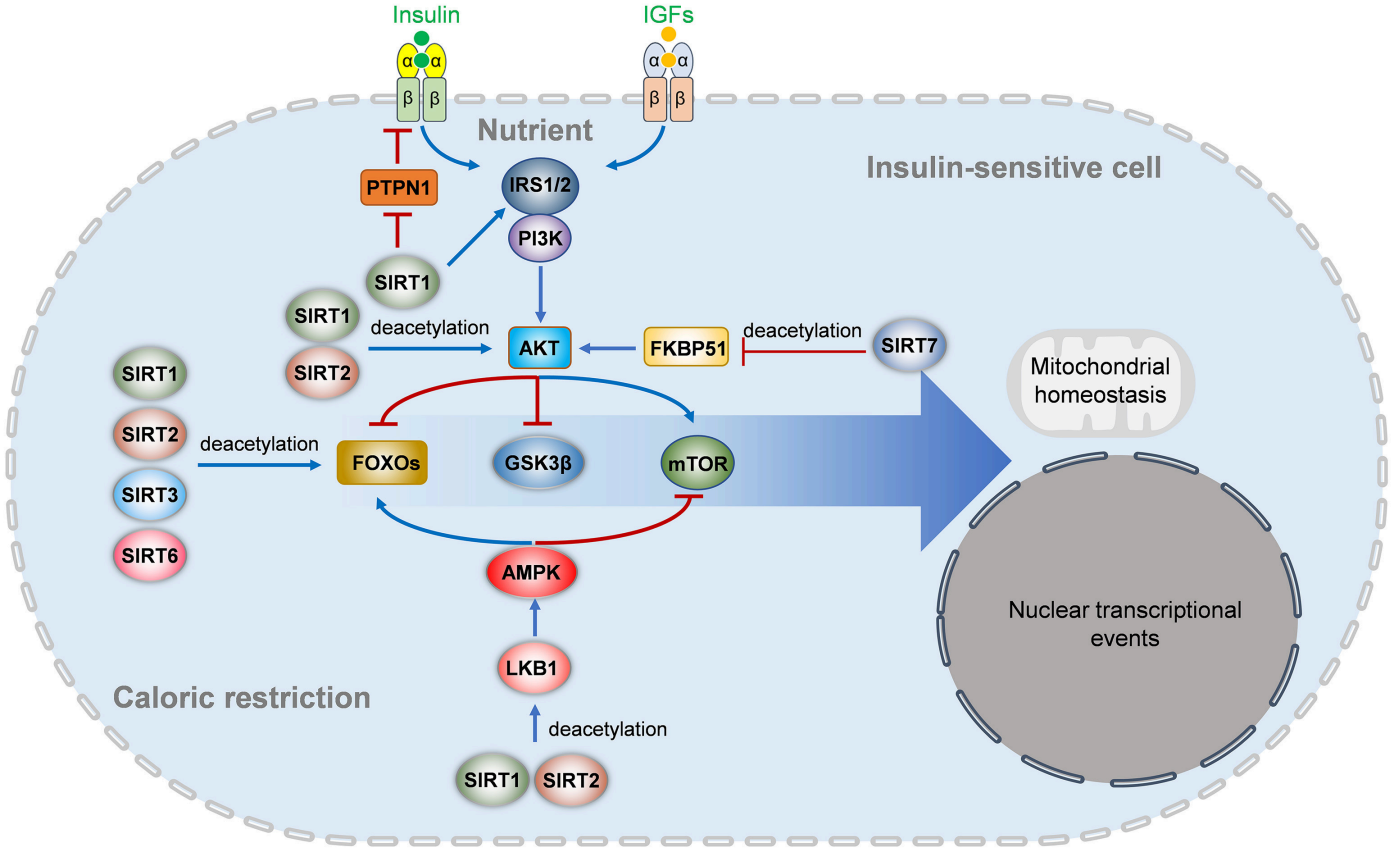
Sirtuins regulates insulin signaling pathways. In nutrient enough conditions, insulin and insulin-like growth factors activate the IRS-PI3K-AKT signaling and downstream FOXO and mTOR signaling to regulate multiple aspects of metabolism, survival, mitochondrial homeostasis, nuclear transcriptional events, and other cellular behaviors. The insulin receptor activation is inhibited by PTPN1, which is repressed by SIRT1. SIRT1 also deacetylates IRS2 and represses IRS1 phosphorylation and PI3K-AKT activation. SIRT1 and SIRT2 also deacetylate AKT to activate its activation directly, while SIRT7 indirectly inhibits AKT activation by deacetylating FKBP51. Under energetic stress or caloric restriction, multiple members of the Sirtuins family are activated. SIRT1, SIRT2, SIRT3, and SIRT6 can directly deacetylate FOXOs (FOXO1 and FOXO3a), while SIRT1 and SIRT2 activate the LKB1-AMPK signaling to activate FOXO and inhibit mTOR signaling. IGF, insulin-like growth factor; IRS, insulin receptor substrate; PTPN1, tyrosine-protein phosphatase non-receptor type 1; PI3K, phosphatidylinositol-4,5-bisphosphate 3-kinase; mTOR, mammalian target of rapamycin; AMPK, AMP-activated protein kinase; LKB1, liver kinase B1; FKBP51, FK506-binding protein 51; GSK3β, glycogen synthase kinase-3 beta.

Insulin resistance, the inability of cells to efficiently respond to a normal dose of insulin, is caused by impaired insulin signaling and postreceptor intracellular defects (4). Insulin binding to its receptor results in IR phosphorylating itself and several intracellular substrates. The phosphorylated substrates interact with intracellular effectors, leading to the activation of the PI3K-Akt pathway, which is responsible for most of the metabolic actions of insulin, and the Ras-MAPK pathway, which controls cell growth and differentiation (4, 142). Impaired Akt activation is a key factor in metabolic disorders involving insulin resistance. Accumulating evidence suggests that Sirtuins participate in insulin signaling in target cells (Figure 2).

SIRT1 positively regulates insulin signaling and Akt activation at multiple levels. SIRT1 represses transcription of *PTPN1*, a negative regulator of the insulin signal transduction cascade, at the chromatin level and improves insulin sensitivity (3). Knockdown of *Sirt1* in 3T3-L1 adipocytes increases phosphorylation of JNK, as well as serine phosphorylation of insulin receptor substrate 1 (IRS-1), which leads to decrease tyrosine phosphorylation of IRS-1, and then inhibit phosphorylation of Akt (39). Inhibition of SIRT1 activity reduces insulin-induced IRS-2 deacetylation, which prevents insulin-induced IRS-2 tyrosine phosphorylation (143). SIRT1 mediates deacetylation of Akt regulates binding of Akt to phosphatidylinositol 3,4,5-trisphosphate (PIP3) which is necessary for Akt membrane localization and activation (144).

SIRT2 can directly regulate the insulin signaling pathway, but its role is controversial. SIRT2 can deacetylate and activate Akt through the Akt/glycogen synthase kinase-3β (GSK3β)/β-catenin signaling pathway, finally resulting in aberrant proliferation and survival of myeloid leukemia cells and epithelial-mesenchymal transition of HCC (145, 146). Interestingly, Ramakrishnan and colleagues showed that SIRT2 is a novel Akt interactor and is required for optimal Akt activation under normal conditions (147). Pharmacological or genetic inhibition of SIRT2 decreases Akt activation in 3T3-L1 preadipocytes and HeLa cells, whereas SIRT2 overexpression enhances the activation of Akt and its downstream targets, such as GSK3 and p70-S6-kinase (147). Insulin-induced Akt activation requires Akt binding to inositol 1,4,5-trisphosphate, which leads to Akt conformational changes and facilitates its phosphorylation by PDK1 and mTORC1 (147). Acetylation at Lys20 blocks Akt activation by restricting the binding of Akt to inositol 1,4,5-trisphosphate (144). However, the authors were unable to detect Akt acetylation in the experiment, and they could not determine whether the effects of SIRT2 on Akt are dependent on changing the acetylation level of Akt (147). However, the opposite results have also been reported. Arora et al. reported that SIRT2 is upregulated in insulin-resistant skeletal muscle cells, and inhibition of SIRT2 by pharmacological or genetic means improves phosphorylation of Akt and GSK3β and increases insulin-stimulated glucose uptake (148). Similarly, in insulin-resistant neuro-2a cells, inhibition of SIRT2 by pharmacological or genetic means also enhances the activity of Akt and increases insulin-stimulated glucose uptake (149). Therefore, SIRT2 may regulate Akt in both direct (activity-dependent) and indirect (activity-independent) manners, which may largely rely on the metabolic status of the cells. SIRT2 can deacetylate and regulate the function of FOxO transcription factors, which are direct Akt targets (98, 150). Interestingly, the Akt-independent pathway also contributes to the function of SIRT2 in the regulation of insulin sensitivity. TUG acetylation modulates its interaction with Golgi matrix proteins and enhances its function to trap GLUT4 storage vesicles in intracellular (151). Insulin mobilizes the exocytic translocation of GLUT4 glucose transporters by triggering TUG proteolysis to accelerate glucose uptake in fat and muscle. SIRT2-mediated TUG deacetylation controls insulin sensitivity *in vivo* and *in vitro* (151).

The role of SIRT6 in the insulin signaling pathway is controversial. *Sirt6*-deficient mice die about 4 weeks of age, exhibiting severe metabolic defects, including low insulin and hypoglycemia (15). Xiao et al. found that *Sirt6* deficiency increases Akt phosphorylation through modulating insulin signaling upstream of Akt, including insulin receptor, IRS1, IRS2, and enhances insulin signaling, leading to hypoglycemia (152). On the contrary, *Sirt6* transgenic mice show increased insulin sensitivity in skeletal muscle and liver and exhibit enhanced insulin-induced Akt activation in gastrocnemius (153).

In addition, SIRT7 can regulate Akt signaling. SIRT7 regulates the acetylation of FKBP51, which then regulates Akt activation. Acetylated FKBP51 enhances Akt activity by blocking its interaction with PHLPP-Akt. SIRT7 deacetylates FKBP51 at two major lysine residues. SIRT7 suppresses Akt activation and modulates cell sensitivity to genotoxic agents (154). The inhibitory effects of SIRT7 on Akt activation were also observed in murine hearts.

## Sirtuins in Insulin-Sensitive Organs

Adipose tissue, liver, and muscle are the primary insulin-responsive organs. Insulin regulates blood glucose concentrations by suppressing hepatic glucose output and stimulating glucose uptake by muscle and adipose tissue. In addition, insulin promotes energy storage in adipose tissue, liver, and muscle by stimulating lipogenesis, glycogen, and protein synthesis but inhibiting lipolysis, glycogenolysis and protein catabolism (155).

The impaired lipogenic/adipogenic capacity of adipose tissue leads to increased body fat mass and adverse metabolic consequences (4, 156). Adipose tissue is not only an excessive energy storage pot but also a highly active endocrine organ that secretes proteins, adipokines, cytokines and chemokines to influence insulin sensitivity. Circulating FFAs derived from adipocytes are involved in the accumulation of triglycerides and fatty acid-derived metabolites in muscle and liver, which is a contributing factor to insulin resistance (155). In addition, adipose tissue is an important initiator of the inflammatory response to obesity (28), and FFAs from adipose tissue are important ER stress-triggering factors (28).

The liver, as the central organ responsible for maintaining lipid and glucose hemostasis in the body, plays a crucial role in insulin sensitivity and metabolic diseases. During prolonged fasting or starvation, the liver converts lipids to available energy through fatty acid oxidation and provides glucose to maintain normal blood glucose, initially by glycogenolysis and then by switching to gluconeogenesis (157, 158). Under energy abundance conditions, the liver promotes glycogenesis and lipogenesis to store energy. Insulin stimulates glycogen accumulation and blocks gluconeogenesis and glycogenolysis in the liver to suppress hepatic glucose output (155, 158, 159). In the condition of insulin resistance, suppression of hepatic glucose output is impaired, while increased FFA from adipocytes leads to ectopic lipid accumulation in the liver, which exacerbates insulin resistance (29).

Skeletal muscle is the major site for insulin-stimulated glucose disposal *in vivo* as well as the main energy consumer of lipid catabolism that strongly influences whole-body lipid metabolism (155, 160). The ability to switch between glucose and lipid oxidation is crucial for skeletal muscle to maintain physiological function and metabolic hemostasis (161). Intramuscular fatty acid metabolite accumulation may cause insulin resistance (162).

**Figure 3:**
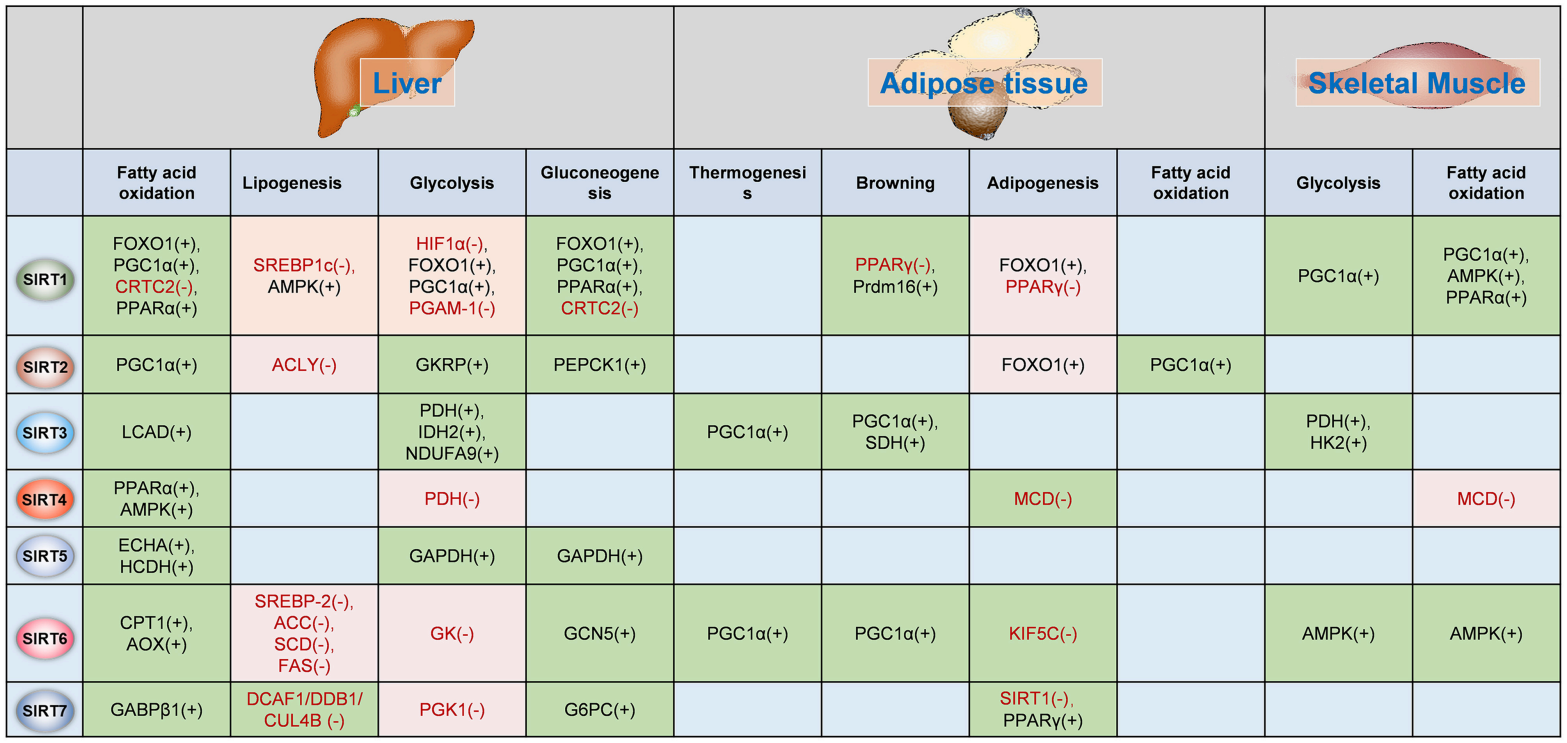
Sirtuins regulates metabolism in insulin-target organs. The functions of Sirtuins in the regulation of glucose and fatty acid metabolism in the liver, adipose tissue, and skeletal muscle. The (–) indicates Sirtuin represses the activation/expression of this target, whereas (+) indicates Sirtuin promotes the activation/expression of the target. The green background indicates Sirtuin promotes the biological process whereas the pink background indicates the Sirtuin represses the biological process. PGC-1α, peroxisome proliferator-activated receptor gamma coactivator 1-alpha; TORC2, CREB regulated transcription coactivator 2; PPARα, peroxisome proliferator-activated receptor alpha; SREBP1c, Sterol response element-binding protein 1c; AMPK, AMP-activated protein kinase; HIF1α, hypoxia-inducible factor 1 alpha; PGAM-1, phosphoglycerate mutase 1; CRTC2, CREB regulated transcription coactivator 2; PPARγ, peroxisome proliferator-activated receptor gamma; Prdm16, PR domain containing 16; PEPCK1, phosphoenolpyruvate carboxykinase 1; LCAD, long-chain acyl-CoA dehydrogenase; PDH, pyruvate dehydrogenase; IDH2, isocitrate dehydrogenase; NDUFA9, NADH dehydrogenase [ubiquinone] 1 alpha subcomplex subunit 9; SDH, succinate dehydrogenase; HK2, hexokinase 2; MCD, malonyl-CoA decarboxylase; ECHA, trifunctional enzyme subunit alpha; HCDH, hydroxyacyl-Coenzyme A dehydrogenase; GAPDH, Glyceraldehyde 3-phosphate dehydrogenase; SREBP-2, sterol regulatory element-binding protein 2; ACC, acetyl-CoA carboxylase; SCD, stearoyl-CoA desaturase; FAS, fatty acid synthase; GK, glucokinase; GCN5, general control non-repressed protein 5; KIF5C, kinesin heavy chain isoform 5C; GABPβ1, GA binding protein β1; DCAF1/DDB1/GUL4B, DDB1-CUL4-associated factor 1 (DCAF1)/damage-specific DNA binding protein 1 (DDB1)/cullin 4B (CUL4B) complex; PGK1, phosphoglycerate kinase 1; G6PC, glucose-6-phosphatase, catalytic subunit; GKRP, glucokinase regulatory protein.

The functions of Sirtuins in regulating glucose and lipid metabolism as well as insulin sensitivity have been widely investigated in adipose tissue, liver and skeletal muscles (Figure 3).

### SIRT1 and Insulin-Sensitive Organs

#### SIRT1 Regulates Fatty Acid and Glucose Metabolism in the Liver

The role of SIRT1 in regulating hepatic gluconeogenesis is controversial under the condition of calorie restriction. Resveratrol has been shown to improve glucose homeostasis in insulin-resistant mice by reducing hepatic gluconeogenesis and increase insulin sensitivity in adipose tissue, skeletal muscle and liver (163). On the contrary, resveratrol causes nuclear translocation of FoxO1 in hepatocytes *via* SIRT1-dependent deacetylation, which leads to activation of gluconeogenesis and increased hepatic glucose output (164). Pyruvate induces SIRT1 protein in the liver during fasting, and once SIRT1 is induced, which can increase gluconeogenic genes expression and promote hepatic glucose output through interacting and deacetylating PGC-1α (165). Conversely, during prolonged fasting, SIRT1 deacetylates CREB regulated transcription coactivator 2 (CRTC2) and promotes its ubiquitin-dependent degradation to inhibit gluconeogenic gene expression, leading to decreased hepatic glucose output (166). On the other hand, upregulation of FOXO1 and PGC-1α activity by SIRT1 leads to activation of gluconeogenic gene expression in hepatic cells and increase hepatic glucose production (157, 166). In addition, SIRT1 regulates the activity of PGC-1α, and glycolytic enzyme phosphoglycerate mutase-1 (PGAM1), inducing repression of glycolytic genes in response to fasting (165, 167). These studies suggest an important role of SIRT1 in maintaining energy balance under fasting. Liver-specific *Sirt1* ko mice develop hepatic steatosis when underwent fasting and obesity when fed with HFD in insulin-dependent and independent manners (38, 168). *Sirt1* transgenic mice show better glucose tolerance and insulin sensitivity and are almost entirely protected from hepatic steatosis with HFD treatment (37, 169). On one hand, SIRT1 deacetylates and activates PGC-1α, increasing fatty acid β-oxidation in the liver (38). On the other hand, SIRT1 plays an important role in inhibiting lipogenesis in the liver. For example, resveratrol can increase the levels of sterol regulatory element-binding protein (SREBP), a critical regulator of lipid and sterol homeostasis in eukaryotes, in livers of alcohol-treated mice and alleviate alcoholic fatty liver (170). SRT1720, a SIRT1 activator, ameliorates fatty liver through suppressing the expression of lipogenic enzymes, including SREBP-1c, acetyl-CoA carboxylase, and fatty acid synthase, in obesity and insulin resistant mice (171). SIRT1 can directly deacetylate SREBP, and control SREBP protein stability *via* SREBP ubiquitination, leading to attenuating SREBP target lipogenic gene expression and inhibiting lipid synthesis and fat storage (172). Otherwise, SIRT1 activation by polyphenols acts as an upstream regulator in the LKB1/AMPK signaling axis, resulting in repression expression of acetyl-CoA carboxylase and fatty acid synthase and reduction of lipid accumulation in hepatocytes (173).

#### SIRT1 Regulates Adipocyte Differentiation and Adipogenesis

PPARγ and CCAAT/enhancer-binding protein α (C/EBPα) are master regulators of adipogenesis (156, 174). SIRT1 overexpression inhibits the expression of PPARγ and C/EBPα in 3T3-L1 adipocytes (175). SIRT1 represses PPARγ by docking with its cofactors nuclear receptor co-repressor (NCoR) and silencing mediator of retinoid and thyroid hormone receptors (SMRT) and suppresses adipogenesis (175). In differentiated fat cells, upregulation of SIRT1 by resveratrol triggers lipolysis and loss of fat, but SIRT1 inhibitor nicotinamide reduces the release of free fatty acid (175).

#### SIRT1 Regulates Lipid Metabolism in Skeletal Muscle

There is a strong correlation between the presence of intramyocellular lipid in skeletal muscle and liver and progression of T2DM (176, 177). Fasting induces PGC-1α deacetylation by SIRT1 in skeletal muscle, and that is required for activation of mitochondrial fatty acid oxidation genes (178). SIRT1 overexpression protects C2C12 myotubes against fatty acid-induced insulin resistance through transcriptional repression of PTP1B (3). In skeletal muscle, SIRT1 acts downstream of AMPK signaling, deacetylates and modulates the activity of PGC-1α, FOXO1, and FOXO3a to inhibit lipogenesis and promote energy consumption (179). SIRT1 overexpression or resveratrol treatment increases insulin-induced Akt phosphorylation and activation *via* interacting with the PI3K adapter subunit p85 (180). These studies suggest SIRT1 plays a positive role in ameliorating insulin sensitivity in skeletal muscle. However, several studies have demonstrated that skeletal muscle-specific overexpression of SIRT1 does not enhance whole-body energy expenditure or skeletal muscle insulin sensitivity under normal or overfeeding conditions (181, 182). These controversial results suggest SIRT1 in other metabolic tissues, such as adipose tissue, liver or intestinal tissue, may play a role in the metabolic benefits of SIRT1 activation (183). Notably, high-intensity interval training increases SIRT1 activity in human skeletal muscle and mice with muscle-specific inactivation of the SIRT1 deacetylase domain displayed reduced myofiber size, impaired muscle regeneration, and derepression of muscle developmental genes (184, 185). Therefore, SIRT1-mediated metabolic balance is important for skeletal muscle homeostasis and regeneration.

### SIRT2 and Insulin-Sensitive Organs

#### SIRT2 Regulates Adipocyte Differentiation and Adipogenesis

SIRT2 is widely distributed and has been detected in a wide range of metabolic tissues, including the brain, muscle, liver, pancreas and adipose tissue. SIRT2 expression is regulated by metabolic status. For instance, the expression of SIRT2 is elevated in the white adipose tissue (WAT) of cr mice (98). SIRT2 gene expression increased in the peripheral blood mononuclear cells of obese subjects following an 8-week hypocaloric diet (186). By contrast, SIRT2 protein expression in visceral WAT from human obese subjects and a mouse model of diet-induced obesity is downregulated compared with that in WAT from lean controls (187). SIRT2 gene expression is significantly lower in peripheral blood mononuclear cells of obese children with insulin resistance than in those without insulin resistance (188). A growing body of literature has indicated that SIRT2 is involved in regulating various metabolic processes, including adipocyte differentiation, hepatic gluconeogenesis, and insulin action. SIRT2 mRNA is more abundant than other Sirtuins in adipose tissue *in vivo* and preadipocytes in culture (150), implicating the possible important role of SIRT2 in adipose tissue. FOXO1 acts as an adipogenesis inhibitor (189). In adipose tissue, FOXO1 can interact with PPARγ and negatively regulate its transcriptional activity (190) or bind to the PPARγ promoter region and suppress its expression (191). In mouse 3T3-L1 preadipocytes, SIRT2 interacts with and deacetylates FOXO1, which antagonizes FOXO1 phosphorylation and promotes nuclear retention of FOXO1, leading to repression of the expression of *PPAR*γ and *C/EBP*α as well as genes marking terminal adipocyte differentiation, such as *Glut4, aP2*, and *fatty acid synthase* (150). SIRT2 also suppresses adipogenesis by deacetylating FOXO1 to promote the binding of FOXO1 to PPARγ and subsequent repression of PPARγ transcriptional activity (192). Increased *de novo* lipogenesis is an important contributor to increased adipose mass (193). ATP-citrate lyase (ACLY) is the building block for *de novo* lipid synthesis, which converts glucose-derived citrate into acetyl-CoA (194). ACLY is acetylated on multiple lysine residues in response to high glucose and promotes lipogenesis, while SIRT2 deacetylates and destabilizes ACLY, leading to reduced lipogenesis (195). Transcriptional regulators such as PPARs and the coactivator PGC-1α play key roles in the process of fatty acid β-oxidation, which determines whole-body energy expenditure (196). Through transcriptional repression of SIRT2, hypoxia-inducible factor 1α (HIF1α) decreases deacetylation of PGC-1α and further diminishes fatty acid β-oxidation in WAT. Adipocyte-specific HIF1α inactivation leads to increased expression of SIRT2 and attenuates dietary-driven obesity in mice (187). These studies suggest that SIRT2 contributes to the control of adipose tissue mass by inhibiting adipogenesis and lipogenesis but promoting fatty acid β-oxidation.

#### SIRT2 Participates in Gluconeogenesis in the Liver

SIRT2 deacetylates and subsequently increases the stability of PEPCK1, the gluconeogenic rate-limiting enzyme under conditions of glucose deprivation, leading to increased gluconeogenesis (197, 198). FOXO1 and PGC-1α reportedly activate the process of gluconeogenesis in the liver by increasing the transcription of gluconeogenic enzyme genes and are considered negative regulators of insulin sensitivity in the liver (199–201). Insulin suppresses gluconeogenesis by regulating the FOXO1-PGC-1α interaction (199). SIRT2 deacetylates and activates FOXO1/PGC-1α in adipocytes (150, 187, 192), which implies that SIRT2 may enhance gluconeogenesis through the FOXO1-PGC-1α pathway. However, whether the role of SIRT2 in gluconeogenesis depends on different nutrient conditions must be elucidated. In addition, the roles of SIRT2 in metabolic diseases are largely unknown.

### SIRT3

Human SIRT3 is expressed in a variety of metabolically active tissues, including muscle, liver, kidney, heart, brain, and BAT (202–204). The expression of SIRT3 in the liver and adipose tissue of mice increases during cr (205–208). A single nucleotide polymorphism in the human *SIRT3* gene has been correlated with the reduced enzymatic efficiency of SIRT3 and the development of metabolic syndrome (209). Compared with WT mice, *Sirt3-*KO mice fed an HFD show accelerated obesity, insulin resistance, hyperlipidemia, and hepatic steatosis (209).

#### SIRT3 Participates in WAT/BAT Metabolism and Thermogenesis

High levels of SIRT3 occur in the BAT. Cold exposure upregulates SIRT3 expression in the BAT. Increasing the expression of PGC-1α and uncoupling protein 1 (UCP1) by sustained expression of SIRT3 in brown adipocytes leads to increased thermogenesis (7, 206). Fatty acid β-oxidation in the BAT of *Sirt3-*KO mice is significantly reduced (210). Although SIRT3 maintains a low level in WAT (206), several studies refer to the role of SIRT3 in regulating WAT lipid metabolism. cr activates SIRT3 expression in both white and brown adipose tissue; SIRT3 expression decreases in the BAT of several lines of genetically obese mice (206). In a human study, SIRT3 gene expression was decreased in VAT from morbid subjects (211) and WAT from children with obesity (212). Although the role of SIRT3 in WAT lipid metabolism is intricate and unclear, these studies suggest that SIRT3 may play a protective role in obesity.

#### SIRT3 Regulates Fatty Acid Oxidation in the Liver

*Sirt3*-deficient mice show higher levels of fatty acid β-oxidation intermediate products and triglycerides in the liver during fasting and develop hepatic steatosis (210). Metabolomic analyses of fasted *Sirt3-*deficient mice revealed that SIRT3 is involved in fatty acid β-oxidation and modulates fatty acid β-oxidation at multiple points, such as short-chain L-3-hydroxy acyl-CoA dehydrogenase (SCHAD), very-long-chain acyl-CoA dehydrogenase (VLCAD) and 3-ketoacyl-CoA thiolase, in addition to LCAD (208). LCAD is a key enzyme in mitochondrial fatty acid β-oxidation, and LCAD deficiency causes hepatic steatosis and hepatic insulin resistance (213, 214). SIRT3 promotes hepatic fatty acid β-oxidation through deacetylation and activation of LCAD (210). However, hepatocyte-specific *Sirt3-*KO mice do not show any obvious metabolic phenotype under either chow or HFD conditions, despite a marked global hyperacetylation of mitochondrial proteins (215). These conflicting findings from global *Sirt3-*KO mice and tissue-specific KO mice suggest that the roles of SIRT3 in other cell types may be important for SIRT3-mediated metabolic effects in the liver.

#### SIRT3 Regulates Glucose Metabolism in Skeletal Muscle

SIRT3 expression decreases in the skeletal muscle of diabetic and HFD-fed mice (36, 216). CR and exercise upregulate SIRT3 expression in mouse skeletal muscle (162). These findings suggest that SIRT3 is involved in skeletal muscle metabolism. Although muscle-specific *Sirt3* KO in mice shows no obvious effects on global metabolic hemostasis under normal conditions (215), striking results have been shown in global *Sirt3-*KO mice. Global *Sirt3-*KO mice exhibit decreased oxygen consumption and enhanced oxidative stress in skeletal muscle that leads to impaired insulin signaling (36). The deletion of *Sirt3 in vivo* and *in vitro* induces hyperacetylation of the pyruvate dehydrogenase (PDH) E1α subunit and leads to decreased PDH enzymatic activity (161). Inhibition of PDH activity reduces glucose oxidation and results in a switch to fatty acid β-oxidation, thus leading to a loss of skeletal muscle metabolic flexibility (161). In addition, HFD-fed *Sirt3-*KO mice exhibit increased insulin resistance due to defects in skeletal muscle glucose uptake (217). These studies suggest that SIRT3 may protect insulin sensitivity in skeletal muscle.

### SIRT4

#### SIRT4 Regulates Lipogenesis

The expression of SIRT4 is upregulated in the liver and adipose tissues in rodents fed an HFD (218, 219). SIRT4 deacetylates and inhibits malonyl CoA decarboxylase (MCD), an enzyme producing acetyl-CoA from malonyl CoA, consequently repressing fatty acid oxidation but promoting lipogenesis in WAT and skeletal muscle under nutrient abundance conditions. *Sirt4*-KO mice display increased exercise tolerance and protection against diet-induced obesity (220).

#### SIRT4 Regulates Fatty Acid Oxidation

SIRT4 inhibition in mouse primary hepatocytes increases fatty acid oxidation gene expression, leading to increased fat oxidative capacity in liver (118). The same result is obtained in muscle (118). Similarly, primary hepatocytes from *Sirt4-*KO mice exhibit higher rates of fatty acid oxidation. SIRT4 suppresses PPARα activity and inhibits hepatic fatty acid oxidation by modulating SIRT1 activity (221). Livers from NAFLD patients exhibit increased SIRT4 and lipogenic gene expression (222). These results support the notion that SIRT4 is likely to inhibit fatty acid oxidation and potentiate ectopic lipid storage in liver and skeletal muscle.

### SIRT5

#### SIRT5 in the Regulation of Fatty Acid Metabolism

SIRT5 is highly expressed in metabolic tissues, including the heart, skeletal muscle, brain, liver, and kidney (121). Using a label-free quantitative proteomic approach, Rardin et al. characterized the lysine succinylome in liver mitochondria and revealed a major role for SIRT5 in regulating many metabolic pathways, including β-oxidation and ketogenesis (223). Park et al. revealed that SIRT5 desuccinylates a set of metabolic enzymes in mitochondria that are involved in amino acid degradation, the TCA cycle and fatty acid metabolism (224). In contrast to the other two mitochondrial Sirtuins, SIRT5 protein levels do not change during CR (121, 207). However, similar to *Sirt3*-KO and *Sirt4-*KO mice, *Sirt5*-KO mice do not show any overt metabolic abnormalities under either normal chow or HFD conditions (225). Sirt5 deficiency does not protect or sensitize mice to the development of HFD-induced obesity, hypertension, and insulin resistance (225). The results from *Sirt5-*KO mice suggest that SIRT5 is not dispensable for cellular metabolism, at least under normal conditions. Subsequent studies have shown promising results. In humans, *SIRT5* gene expression decreases in the liver of NAFLD patients (222), and the expression of *SIRT5* in adipose tissue is positively correlated with insulin sensitivity (226). Using affinity enrichment and label-free quantitative proteomics, Nishida *et al*. characterized the SIRT5-regulated lysine malonylome (227). Pathway analysis identified gluconeogenesis and glycolysis as the pathways most enriched in SIRT5-regulated malonylated proteins (227). SIRT5 regulates glyceraldehyde phosphate dehydrogenase (GAPDH), a glycolytic enzyme, through demalonylation of lysine 184 (227). According to these results, SIRT5 may play a critical role in regulating glucose and lipid metabolism and preserving insulin sensitivity. Mitochondria-specific Sirtuin knockout mice show no obvious metabolic abnormalities, indicating that mitochondrial Sirtuins serve as nutrient sensors to maintain energy homeostasis.

### SIRT6 and Insulin-Sensitive Organs

#### SIRT6 Regulates Adipogenesis, Lipid Metabolism and Thermogenesis in Adipose Tissue

SIRT6 expression is decreased in adipose tissue of *db/db* mice but increased in adipose tissue of human individuals with weight loss (228, 229), suggesting that SIRT6 plays a role in adipose tissue. Chen et al. (230) demonstrated that SIRT6 is required for mitotic clonal expansion during adipogenesis by inhibiting expression of kinesin family member 5C (KIF5C) and subsequent increasing CK2 kinase activity. *Sirt6* transgenic mice exhibit resistance to HFD-induced obesity and insulin resistance (231). Conversely, fat-specific *Sirt6* knockout increases blood glucose levels and hepatic steatosis, and sensitizes mice to HFD-induced obesity and insulin resistance (59, 60, 232). SIRT6 overexpression downregulates a set of PPARγ target genes that are involved in lipid metabolism, lipid transport and adipogenesis (231). Especially, SIRT6 decreases expressions of ANGPTL4, a negative regulator of lipoprotein lipase, and diglyceride acyltransferase 1 (DGAT1), a key enzyme in triglycerides synthesis, leading to the increased serum triglyceride clearance and reducing triglyceride synthesis in adipose tissues (231). *Sirt6* deletion decreases FoxO1 transcriptional activity by increasing its acetylation and phosphorylation and reduces expression of adipose triglyceride lipase (ATGL), a key lipolytic enzyme, reducing lipolysis (59). Fat-specific *Sirt6* knockout not only induces obesity and insulin resistance but also impairs the thermogenic function of brown adipocytes (232). Yao et al. (232) found *Sirt6* deletion decreases ATF2 binding to the PGC-1α promoter, leading to reducing the expression of PGC-1α and PGC-1α target thermogenic genes.

#### SIRT6 Represses Gluconeogenesis and Lipid Accumulation in the Liver

The hepatic SIRT6 level is reduced in obese/diabetic mice and gluconeogenic genes were higher in *Sirt6-*deficient livers whereas ectopic re-expression of SIRT6 suppressed gluconeogenesis and normalizes glycemia (228, 233). Mechanistically, SIRT6 interacts with and increases the activity of general control non-repressed protein 5 (GCN5), an acetyltransferase, which, in turn, catalyzes the acetylation of PGC-1α, suppressing gluconeogenic gene expression such as *phosphoenolpyruvate carboxykinase C (PEPCK-C)* and *glucose 6-phosphatase*, and resulting in repression of hepatic glucose output (228). p53 directly activates expression of SIRT6, which subsequently interacts with and deacetylates FoxO1, leading to FoxO1 export to the cytoplasm, and finally, reduce the expression of gluconeogenetic genes such as *glucose 6-phosphatase alpha* and *phosphoenolpyruvate carboxykinase 1* (234). Human fatty liver samples exhibited significantly lower levels of SIRT6 than normal controls and liver-specific deletion of *Sirt6* in mice causes increased glycolysis, triglyceride synthesis, reduced β-oxidation, and leads to liver steatosis (235). Rosiglitazone, an agonist of PPARγ, increases the expression of SIRT6, PGC-1α, and FoxO1, and AMPK phosphorylation in rat liver and ameliorates hepatic lipid accumulation (236). *Sirt6* knockdown abolished the effects of rosiglitazone (236), suggesting *Sirt6* at least partly mediates the metabolic effects of rosiglitazone. Altogether, those evidence suggest that SIRT6 significantly participates in glucose and lipid metabolism in the liver.

#### SIRT6 Increases Insulin Sensitivity in the Skeletal Muscle

SIRT6 also regulates metabolic homeostasis in the skeletal muscle. *Sirt6* transgenic mice show enhanced insulin sensitivity in skeletal muscle and exhibit enhanced insulin-induced activation of Akt in the gastrocnemius (153). By contrast, skeletal muscle-specific *Sirt6* ko mice exhibit impaired glucose homeostasis and insulin sensitivity, attenuating whole-body energy expenditure (237). Mechanistically, *Sirt6* deletion decreases AMPK activity and subsequently decreases the expression of genes involved in glucose and lipid uptake, fatty acid oxidation, and mitochondrial oxidative phosphorylation (237). Further studies are needed to elucidate the direct mechanism underlying SIRT6 function in skeletal muscle.

### SIRT7

#### SIRT7 Regulates Fatty Acid Metabolism in Adipose Tissues

SIRT7 is the least characterized Sirtuin of the seven mammalian Sirtuins. SIRT7 protein levels are high in the liver, spleen, and testis, whereas are low in the muscle, heart, and brain of mice (16). In human, *Sirt7* mRNA is expressed in various tissues (10). Recent reports clarify the important roles of SIRT7 in a variety of biological processes including DNA repair, chromatin assembly, and aging. However, the role of SIRT7 in metabolism remains largely unknown. The expression of *Sirt7* mRNA level is upregulated in adipose tissues of obese patients (238). In HFD-fed mice, *Sirt7* knockout decreased the expression of the fatty acid transporter CD36 in WAT (239). In addition, *Sirt7* knockout led to an increase of thermogenesis along with increased expression of UCP1 and DIO2 in BAT (239). These results suggest SIRT7 regulates lipid metabolism in adipocytes. Recently, Fang *al et*. found that SIRT7 restricts SIRT1 activity by preventing SIRT1 auto-deacetylation, and increasing SIRT1 activity in *Sirt7*-KO mice blocks PPARγ and adipocyte differentiation, thereby decreases the accumulation of white fat (240, 241). Together, these findings implicate the important role of SIRT7 in the regulation of fatty acid metabolism.

#### SIRT7 Regulates Fatty Acid and Glucose Metabolism in the Liver

Up to now, there are three studies linking SIRT7 to the liver lipid metabolism using independently generated mouse models. Shin et al. reported that *Sirt7-*KO mice developed steatosis resembling human fatty liver disease (242). Selectively overexpression of SIRT7 in the liver of *Sirt7*-KO mice *via* adeno-associated virus 8 (AAV8)-mediated gene transfer prevents the development of fatty liver (242). The authors found expressions of inflammatory markers and lipogenic genes are increased in *Sirt7-*deficient livers, and they clarified the underlying mechanism as SIRT7 repressing the expression of ribosomal proteins through decreasing Myc activity and further suppressing ER stress (242). Ryu et al. generated a different *Sirt7-*KO mouse by deleting exons 6-9, and observed more general metabolic defects including hepatic microvesicular steatosis, increased blood lactate levels, reduced exercise performance, cardiac dysfunction and age-related hearing loss induced by multisystemic mitochondrial dysfunction (127). Mechanistically, SIRT7 deacetylates GABPβ1, thereby enables it to form the transcriptionally active GABPα/GABPβ heterotetramer, and then promotes mitochondria function (127). Another study has the opposite result. Yoshizawa *et al*. reported that *Sirt7*-KO mice, deleting exons 4-9, are resistant to HFD induced fatty liver, obesity, and glucose intolerance (239). TR4 is a nuclear receptor involved in lipid metabolism and its target genes increase fatty acid uptake and triglyceride synthesis and storage (243). Hepatic SIRT7 was reported to increase TR4 expression through binding with DCAF1/DDB1/CUL4B E3 ubiquitin ligase complex and inhibiting TR4 degradation (239). It is difficult to explain the divergence of three *Sirt7-*KO mouse models with different genetic background. Liver-specific knockout or *Sirt7* transgene mouse model may be helpful to clarify the role of SIRT7 in liver lipid metabolism (239, 242). In addition to lipid metabolism, SIRT7 is involved in glucose metabolism. SIRT7 regulates acetylation at the K323 site of phosphoglycerate kinase 1 (PGK1), an important enzyme in glycolysis, decreases PGK1 enzyme activity and inhibits glycolysis in liver cancer cells (244). Yoshizawa *et al*. Found that *Sirt7-*KO mice show decreased expression of the hepatic glucose-6-phosphatase catalytic subunit (G6PC), a key gluconeogenic enzyme, and resistance to glucose intolerance (239). Mechanistically, glucose deprivation stimulates SIRT7 binding to the promoter of G6PC, and deacetylating H3K18 in the G6PC promoter, which results in elevated G6PC expression and promotion of hepatic gluconeogenesis (245).

## Sirtuins in Aging-related Metabolic Defects

Aging is a complex process accompanied by the declines in basal metabolic rate and physical activity. Aging is one of the major risk factors contributing to the development of insulin resistance, obesity, T2DM and metabolic syndrome (246). During the aging process, chronic inflammation and mitochondria dysfunction in pancreatic β cells and insulin-sensitive organs have been demonstrated to be major mechanisms linking aging and insulin resistance (247–250). As mentioned above, Sirtuins play important roles in regulating inflammation and mitochondria function. Sirtuins are critically involved in lifespan and healthspan. Deficiency of Sirtuins (SIRT1, SIRT6, and SIRT7) is associated with shortened lifespan and metabolic diseases (251). Our recent evidence also demonstrated that SIRT2 deficiency also facilitated the aging-related development of cardiac dysfunction, including hypertrophy and fibrosis (252). By contrast, germline or cell-specific overexpression of SIRT1 or SIRT6 were reported to expand lifespan and defense metabolic diseases in insulin-dependent and independent manners (253–255).

Sirtuin-targeted strategies show promising in repressing aging-related insulin resistance and metabolic diseases. For instance, the SIRT1 activator SRT1720 extends lifespan and improves the health of mice fed a standard diet (256, 163). It is well established that caloric restriction (CR), the Sirtuin activator, is an effective and reliable means to defense against aging and extend the lifespan and healthspan of mammals, including monkeys (257). Activation of vascular SIRT1 by CR leads to the repressing of aging-related metabolic vascular diseases, including atherosclerosis and aortic aneurysm (258–260). In human studies, CR also can reduce insulin resistance significantly and delay the onset of metabolic diseases (261, 262). Although the mechanisms by which CR extend lifespan are not fully understood, Sirtuins have been implicated to mediate beneficial effects of CR on aging (263). Notably, the CR mimetics (metformin, resveratrol, rapamycin) could expand lifespan and repress diseases related to insulin resistance in rodents partially through activation of Sirtuins (257, 264). Our data showed that SIRT2 contributes to the effects of metformin on aging-related diseases, including cardiac remodeling (252). Currently, clinical trials investigating the anti-aging effects of metformin is undergoing (ClinicalTrials.gov Identifier: NCT02432287).

Importantly, the Sirtuins do not function in individual metabolic organs or cell types alone during aging. Instead, the Sirtuins orchestrate the crosstalk between different organs or between different cell types within the local microenvironmental niche to maintain metabolic homeostasis and prevent against insulin resistance. The SIRT1 activator SRT3025 provides atheroprotection in *Apoe*^−/−^ mice by reducing hepatic *Pcsk9* secretion and enhancing *Ldlr* expression (265). Resveratrol activates duodenal SIRT1 to initiate a gut-brain-liver neuronal axis that improves hypothalamic insulin sensitivity in rats (183). SIRT1 in intestinal stem cells also contributes to the protection roles of caloric restriction on aging (266). In addition, SIRT3 activation by nitrite and metformin improves insulin sensitivity in skeletal muscle and normalizes pulmonary hypertension associated with heart failure with preserved ejection fraction (267). Sirtuins also regulate inflammatory cells within the local microenvironmental niches to regulate insulin resistance in an autocrine or paracrine manner (41, 79, 80).

Therefore, targeting Sirtuins could be a promising strategy for improvement of insulin sensitivity and metabolic status of the whole body. However, activation of Sirtuins alone may not archive the biggest benefits because of the exhaustion of the endogenous NAD. Sirtuin activator in supplement with NAD precursor may represent a better therapeutic strategy for repressing aging-related insulin resistance and metabolic diseases.

## Concluding Remarks

Insulin resistance is a critical pathological feature of obesity and metabolic syndrome and plays a key role in the pathogenesis of T2DM and attendant cardiovascular complications. Moreover, insulin resistance provides a therapeutic strategy to prevent, delay or treat T2DM, obesity, and metabolic syndrome by improving insulin sensitivity. Although insulin resistance is a complex metabolic disorder that has remained poorly understood, Sirtuin family members are involved in the potential cellular mechanisms of the pathogenesis of insulin resistance. According to accumulating evidence in the past decades, Sirtuin family members have emerged as a nutrient sensor to maintain energy homeostasis. Cellular and animal studies have demonstrated that Sirtuins play an important role in regulating glucose and lipids by modulating crucial enzymes in metabolic pathways and interfering with inflammation, oxidative stress, mitochondrial dysfunction, ER stress, and the insulin signaling pathway.

Sirtuins respond to environmental (diet and lifestyle) or metabolic (obesity, fasting, and diabetes) insults at mRNA and protein levels in insulin-sensing organs (268, 269). The roles of Sirtuins in regulating glucose and lipid metabolism as well as insulin resistance in liver, adipose tissue, and skeletal muscle make their importance in regulating metabolic diseases, including T2DM and diabetic complications (269).

Nevertheless, many additional studies are needed.

1. Different Sirtuins may have the same downstream targets, such as FoxO3a, FoxO1, PGC-1α, and GDH, and there is cross-talk among Sirtuin family members (117, 56, 270). How do different Sirtuin members coordinate to regulate the same downstream targets?
2. The Sirtuin family comprises NAD^+^-dependent histone deacetylases; however, recent results have revealed that Sirtuin members can act in a deacetylase-independent manner (117). How can we determine the functions and activities of Sirtuins in addition to their deacetylation function in insulin resistance?
3. In addition to Sirtuins, there are other epigenetic modification enzymes, including SUV39H1 and EZH2 are involved in insulin resistance and T2DM (271–276). There is an interaction between SUV39H1 and Sirtuins, including SIRT1, SIRT3, and SIRT7 (277–281). EZH2 is reportedly the deacetylating substrate of SIRT1 (282, 283). SIRT2 negatively regulates JMJD2A expression in human non-small cell lung cancer tissues (284). Is JMJD2A involved in insulin resistance? In the condition of insulin resistance, how do these epigenetic modification enzymes influence each other and consequently act on insulin sensitivity?
4. Obesity, insulin resistance, and T2DM are aging-related abnormalities. Sirtuins, especially SIRT1, SIRT2, and SIRT6, are characterized as protectors of aging and aging-related diseases. Whether the promotive effect of aging by the decline of Sirtuin activity is involved in insulin resistance deserves further investigation. In addition, cell senescence-induced organ dysfunction and aging (senescaging) are common during physiological and pathological aging processes (268). Selective elimination of senescent cells, or senolysis, was reported to delay aging and aging-related metabolic diseases including congestive decline, atherosclerosis, cardiac diseases, and osteoarthritis (285–291). However, it remains to elucidate that what are the physiological and pathological functions of cellular senescence in organs during aging and that whether Sirtuins regulate senescaging in insulin resistance and healthy conditions.

## Author Contributions

All authors listed have made a substantial, direct and intellectual contribution to the work. SZ is responsible for literature collection and article draft. XT designed the Figures and revised the manuscript. H-ZC is the leading principal investigator who directed the study and data analysis, and prepared the manuscript. All authors approved publication of this work.

### Conflict of Interest Statement

The authors declare that the research was conducted in the absence of any commercial or financial relationships that could be construed as a potential conflict of interest.
